# Association of Polyvascular Disease and Elevated Interleukin-6 With Outcomes in Acute Ischemic Stroke or Transient Ischemic Attack

**DOI:** 10.3389/fneur.2021.661779

**Published:** 2021-04-13

**Authors:** Ye Tian, Jing Jing, Huijuan Wang, Anxin Wang, Yijun Zhang, Yong Jiang, Jinxi Lin, Xingquan Zhao, Hao Li, Yongjun Wang, Li Guo, Xia Meng

**Affiliations:** ^1^Department of Neurology, The Second Hospital of Hebei Medical University, Shijiazhuang, China; ^2^Department of Neurology, Beijing Tiantan Hospital, Capital Medical University, Beijing, China; ^3^China National Clinical Research Center for Neurological Diseases, Beijing, China

**Keywords:** polyvascular disease, interleukin 6, ischemic stroke, transient ischemic attack, stroke recurrence

## Abstract

**Background:** Polyvascular disease (PolyVD) and interleukin (IL)-6 are associated with poor outcomes in patients with stroke respectively. However, whether combined PolyVD and elevated IL-6 levels would increase the risk of poor outcomes of stroke patients is yet unclear.

**Methods:** Data were obtained from the Third China National Stroke Registry (CNSR-III). PolyVD was defined as acute ischemic stroke (AIS) or transient ischemic attack (TIA) with coronary artery disease (CAD) and/or peripheral artery disease (PAD). Patients were divided into four groups according to the combination of vascular beds number (non-PolyVD or PolyVD) and IL-6 levels (IL-6 < 2.64 pg/mL or IL-6 ≥ 2.64 pg/mL). The primary outcome was a recurrent stroke at 1-year follow-up. Cox proportional hazard models were employed to identify the association of the combined effect of PolyVD and IL-6 with the prognosis of patients.

**Results:** A total of 10,773 patients with IL-6 levels and 1-year follow-up were included. The cumulative incidence of recurrent stroke was 9.87% during the 1-year follow-up. Compared to non-PolyVD and IL-6<2.64 pg/mL patients, patients had non-PolyVD with IL-6 ≥ 2.64 pg/mL (HR 1.245 95%CI 1.072–1.446; *P* < 0.001) and PolyVD with IL-6 <2.64 pg/mL (HR 1.251 95%CI 1.002–1.563; *P* = 0.04) were associated with an increased risk of recurrent stroke during 1-year follow-up. Likewise, patients with PolyVD and IL-6 ≥ 2.64 pg/mL (HR 1.290; 95% CI 1.058–1.572; *P* = 0.01) had the highest risk of recurrent stroke at 1-year follow-up among groups.

**Conclusion:** PolyVD and elevated IL-6 levels are both associated with poor outcomes in patients with AIS or TIA. Moreover, the combination of them increases the efficiency of stroke risk stratification compared with when used alone. More attention and intensive treatment should be given to those patients with both PolyVD and elevated IL-6 levels.

## Introduction

Stroke, a major health concern, is one of the leading causes of mortality and disability worldwide in recent decades (1–4). Despite the current secondary prevention strategies, the recurrence rate of stroke is high (5–9). Atherosclerosis is the main cause of ischemic stroke and chronic systemic disease with a variety of clinical manifestations. Moreover, an atherosclerotic disease involving more than two vascular beds is known as polyvascular disease (PolyVD), and the most common manifestations are coronary artery disease (CAD), cerebrovascular disease (CVD), and peripheral artery disease (PAD) (10). Previous studies have shown that compared to patients with single vascular bed involvement, patients with PolyVD were at a higher risk of major adverse cardiovascular events (MACEs), and this risk elevated with a rise in the number of vascular bed involvement (11, 12). However, poor outcomes of atherosclerotic diseases were not only affected by the number of vascular bed injuries, but also closely related to the stability of plaques.

Inflammatory factors are considered potential risk factors for atherosclerosis development and related to the instability of plaques in ischemic stroke patients (13, 14). Interleukin-6 (IL-6) is an upstream inflammatory factor that plays a critical role as a mediator in propagating the inflammatory response and atherosclerosis progression (15). Accumulating evidence confirmed that IL-6 was associated with adverse outcomes of ischemic stroke (16–18). Although both PolyVD and IL-6 are considered risk factors for poor outcomes of ischemic stroke, there were limited data on their combined effect which might raise the risk stratification in stroke patients.

Thus, the present study used the data of the Third China National Stroke Registry (CNSR-III) to investigate the combined effect of atherosclerosis vascular beds involvement numbers and IL-6 levels on the outcomes in patients with acute ischemic stroke (AIS) or transient ischemic attack (TIA).

## Methods

### Study Design and Population

Data in this study were collected from CNSR-III, a nationwide, prospective registry enrolling consecutive patients with AIS or TIA between August 2015 and March 2018 from 201 hospitals across 22 provinces in China. A total of 15,166 participants with AIS or TIA were enrolled within 7 days of symptom onset. Medical records and biological samples were collected through a standardized process and supervised by an independent Contract Research Organization throughout the study period. The rationale description of the CNSR-III have been illustrated previously (19). This study was approved by the Ethics Committees of Beijing Tiantan Hospital and all other participating centers. Written informed consent was obtained from all participants or their legal representatives before enrollment in the study.

### Data Collection

The baseline data were collected by an electronic data capture system in accordance with a standard collection protocol encompassing age, gender, education level, current smoking, heavy drinking (≥20 g/day), medical history of hypertension, diabetes mellitus (DM), dyslipidemia, atrial fibrillation (AF), previous ischemic stroke (IS), infection (upper respiratory tract, urinary tract, or digestive tract history within 2 weeks before admission), severity of stroke on admission (National Institutes of Health Stroke Scale, NHISS), the etiology classification of ischemic stroke conducted by the centralized TOAST (Trial of Org 10,172 in Acute Stroke Treatment) criteria (20), medication on admission, including antihypertensive drugs, glucose-lowing drugs, antiplatelet drugs, and lipid-lowing drugs, systolic blood pressure (SBP), diastolic blood pressure (DBP), body mass index (BMI), and biochemical markers like glucose, total cholesterol (TC), triglyceride (TG), low-density lipoprotein (LDL), high-density lipoprotein (HDL) and IL-6 levels.

PolyVD was defined as AIS or TIA patients with a history or new diagnosis of CAD and/or PAD in this study. Non-PolyVD was defined as AIS or TIA patients without history and newly diagnosed with CAD or PAD according to the medical history or discharge diagnosis. CAD was diagnosed based on a history of stable angina, unstable angina, myocardial infarction, percutaneous coronary intervention, and coronary artery bypass graft surgery, and/or patients with CAD symptoms confirmed by electrocardiography, echocardiography, and laboratory tests. PAD was diagnosed based on a history of intermittent claudication, peripheral vascular surgery, and intervention, such as angioplasty, stenting, atherectomy, peripheral arterial bypass graft, or other vascular interventions, such as amputation, and/or current intermittent claudication with an ankle brachial index (ABI) <0.9.

### Measurement of IL-6

Fasting blood samples were collected in EDTA anticoagulation blood collection tube within 24 h of admission. Subsequently, plasma samples were collected and frozen in cryotubes at −80°C and transported to the Central Laboratory in Beijing Tiantan Hospital, where all specimens were stored at −80°C until testing. The level of circulating IL-6 was measured in EDTA-plasma samples using ELISA Kit (R&D Systems, Emeryville, CA, USA), according to the manufacturer's guidelines. The laboratory personnel were blinded to the serum specimens, clinical information, and outcomes.

### Follow-Up and Outcomes Assessment

The patients included in this study were followed for clinical outcomes at 1 year *via* telephone by well-trained research coordinators. The primary efficacy outcome was a recurrent stroke during the 1-year follow-up, which was defined as new onset of focal neurological deficits, such as cerebral ischemic or hemorrhagic events. These deficiencies were verified by computed tomography (CT)/magnetic resonance imaging (MRI). The secondary efficacy outcomes included MACEs (IS, hemorrhagic stroke, myocardial infarction, or vascular death), all-cause mortality (death from all causes), and poor functional outcomes defined as modified Rankin scale (mRS) score 3–6 within 1 year.

### Statistical Analysis

Participants were divided into four groups according to different combinations of vascular bed involvement numbers (non-PolyVD or PolyVD) and IL-6 levels (IL-6 < 2.64 pg/mL or IL-6 ≥ 2.64 pg/mL). Since there was no definite target recommended by guidelines currently, the median of IL-6 was considered as the cutoff value in this study (17).

Continuous variables were described as mean ± standard deviation (SD) or median with interquartile ranges (IQR) according to the variable distribution, while categorical variables were presented by frequencies and percentages. The baseline characteristics in different groups were compared by non-parametric Wilcoxon or Kruskal–Wallis test for continuous variables and chi-square tests or Fisher's exact test for categorical variables. The cumulative incidence of outcomes during the follow-up period in different groups was analyzed by Kaplan–Meier curves. Statistical significance was considered as a two-tailed value of *P* < 0.05.

Cox proportional hazard models were employed to investigate the association of combined effect of vascular beds involvement numbers and IL-6 levels, and the risk of recurrent stroke, MACEs, all-cause mortality and poor functional outcomes at 1-year follow-up in an unadjusted or adjusted model. Then, the adjusted hazard ratios (HR) and their 95% confidence intervals (CIs) were calculated. In model 1, we adjusted age, gender, education, current smoking status, heavy drinking, medical history of hypertension, DM, dyslipidemia, IS, AF, NIHSS at admission, the subtype of stroke, medication, and infection. In model 2, we adjusted the variables in model 1 plus variables P <0.05 in the four-group comparison including DBP (mmHg), BMI (mean ± SD), glucose (mmol/L),TC (mmol/L), TG (mmol/L), LDL (mmol/L), and HDL (mmol/L).

All analyses were performed using SAS software version 9.4 (SAS Institute Inc., Cary, NC, USA).

## Results

### Baseline Characteristics

**Figure 1:**
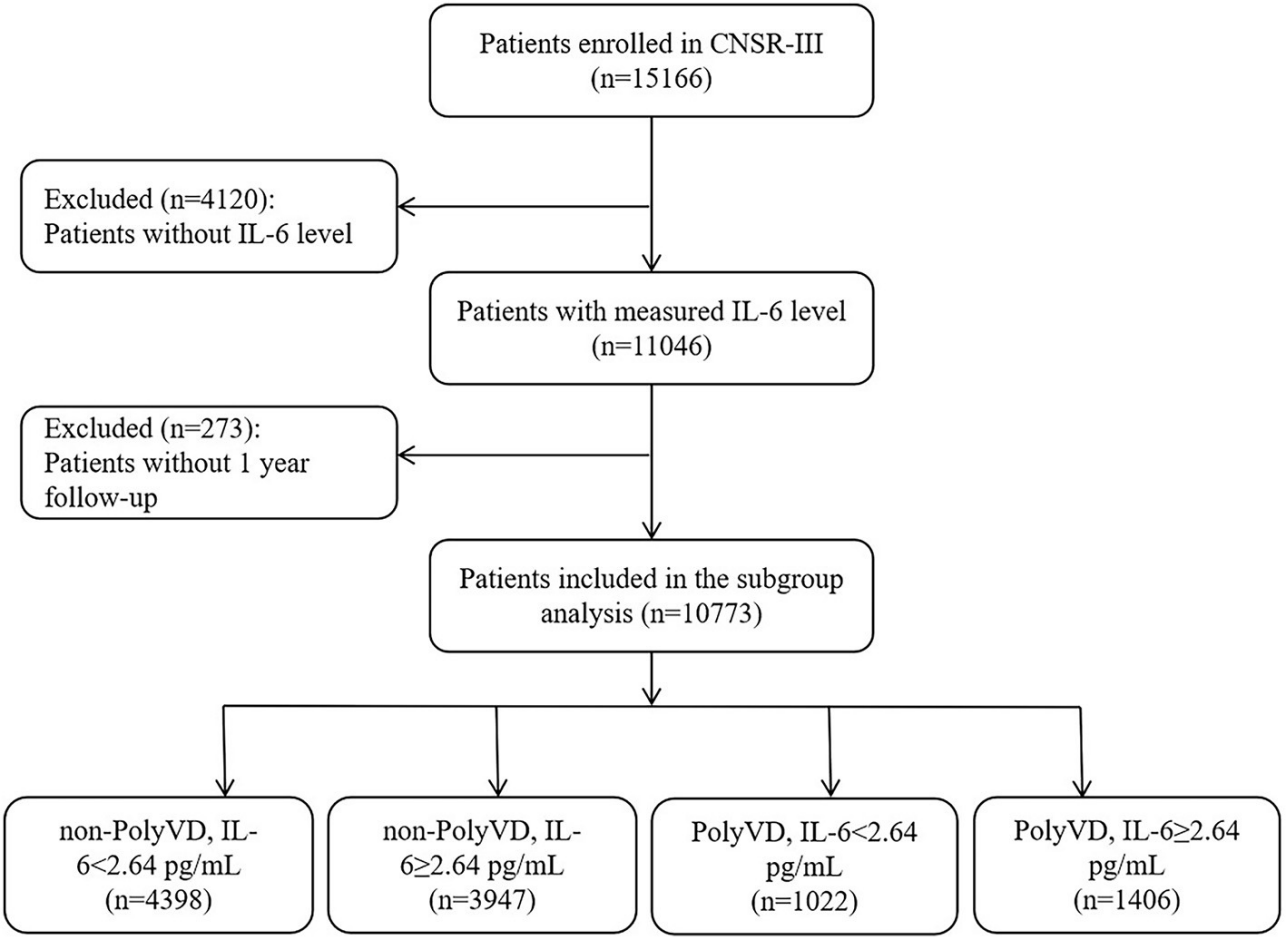
Patient flow diagram. CNSR-III, China National Stroke Registry III; IL-6, interleukin-6; PolyVD, polyvascular disease.

A total of 15,166 AIS or TIA patients were enrolled in the CNSR-III. After the exclusion of patients without IL-6 levels (*n* = 4,120) and 1-year follow-up data (*n* = 273), 10,773 patients were finally included in this study (Figure 1). The baseline characteristics of patients included were as follows: patients included had slightly high education levels, the proportion of DM, dyslipidemia, AF, previous IS, and medication at admission, and slightly low levels of TC, LDL, and HDL (Supplementary Table 1).

Among the 2,428 (22.53%) patients with PolyVD included in this study, the median level of IL-6 was 2.64 pg/ml, and 5,353 (49.69%) patients showed elevated IL-6 levels (≥2.64 pg/ml). Patients were divided into four groups according to different combinations of vascular bed involvement numbers and IL-6 levels. Among the 10,773 included patients, 1,406 (13.05%) had PolyVD with elevated IL-6 levels, while 1,022 (9.49%), 3,947 (36.64%), and 4,398 (40.82%) had PolyVD with non-elevated IL-6 levels, non-PolyVD with elevated IL-6 levels, and non-PolyVD with non-elevated IL-6 levels, respectively.

The demographics and clinical characteristics of patients in different groups are summarized in Table 1. The cohort comprised of 7,376 (68.47%) males, and the mean age was 62.28 ± 11.31 years. Compared to patients with non-PolyVD and IL-6 < 2.64 pg/ml (group 1), patients with PolyVD and IL-6 ≥ 2.64 pg/ml (group 4) were older, had more females, smoked and drank less, and had higher proportions of hypertension, DM, dyslipidemia, AF, previous IS, infection, NIHSS score, and medication usage. In addition, they also showed higher proportions of large-artery atherosclerosis (LAA) and cardioembolism (CE) but a lower proportion of small-vessel occlusion (SVO) in TOAST types. Moreover, the glucose level was higher, while TC, TG, LDL, and HDL levels were lower in group 4 compared to group 1.

**Table 1:** Baseline characteristics of patients according to atherosclerosis vascular beds involvement numbers and IL-6 level.

**Characteristics**	**Total number**	**Overall (*n* = 10,773)**	**non-PolyVD, IL-6 <2.64 pg/ml (*n* = 4,398)**	**non-PolyVD, IL-6 ≥ 2.64 pg/ml (*n* = 3,947)**	**PolyVD, IL-6 <2.64 pg/ml (*n* = 1,022)**	**PolyVD, IL-6 ≥ 2.64 pg/ml (*n* = 1,406)**	***P*-value**
Age (mean ± SD), y	10,773	62.28 ± 11.31	59.02 ± 10.81	63.70 ± 11.43	63.21 ± 9.79	67.80 ± 10.41	<0.0001
Male sex, *n* (%)	10,773	7,376 (68.47)	3,093 (70.33)	2,711 (68.69)	664 (64.97)	908 (64.58)	<0.0001
Education	10,773						<0.0001
Elementary or below		2,919 (27.10)	1,111 (25.26)	1,165 (29.52)	247 (24.17)	396 (28.17)	
Middle school		3,158 (29.31)	1,317 (29.95)	1,120 (28.38)	324 (31.70)	397 (28.24)	
High school or above		3,188 (29.59)	1,390 (31.61)	1,068 (27.06)	323 (31.60)	407 (28.95)	
Unknown		1,508 (14.00)	580 (13.19)	594 (15.05)	128 (12.52)	206 (14.65)	
Current smoking	10,773	3,404 (31.60)	1,472 (33.47)	1,251 (31.69)	296 (28.96)	385 (27.38)	<0.0001
Heavy drinking	1,0773	1,531 (14.21)	647 (14.71)	573 (14.52)	138 (13.50)	173 (12.30)	0.1208
Hypertension	1,0773	6,743 (62.59)	2,576 (58.57)	2,440 (61.82)	724 (70.84)	1,003 (71.34)	<0.0001
Diabetes mellitus	1,0773	2,586 (24.00)	907 (20.62)	933 (23.64)	325 (31.08)	421 (29.94)	<0.0001
Dyslipidemia	10,773	905 (8.40)	315 (7.16)	314 (7.96)	116 (11.35)	160 (11.38)	<0.0001
Atrial fibrillation	10,773	762 (7.07)	108 (2.46)	323 (8.18)	85 (8.32)	246 (17.50)	<0.0001
Previous ischemic stroke	10,773	2,432 (22.57)	815 (18.53)	919 (23.28)	258 (25.24)	440 (31.29)	<0.0001
Infection, *n* (%)	1,0773	308 (2.86)	91 (2.11)	126 (3.12)	30 (2.97)	61 (4.30)	<0.0001
NIHSS score at admission, median (IQR)	10,773	3 (1–6)	3 (1–5)	4 (2–7)	3 (1–5)	4 (2–8)	<0.0001
Subtype of stroke (TOAST)	10,773						<0.0001
LAA		2,692 (24.99)	873 (19.85)	1,182 (29.95)	231 (22.60)	406 (28.88)	
CE		684 (6.35)	142 (3.23)	288 (7.30)	83 (8.12)	171 (12.16)	
SVO		2,258 (20.96)	1,138 (25.88)	715 (18.12)	203 (19.86)	202 (14.37)	
Other causes		5,139 (47.70)	2,245 (51.05)	1,762 (44.64)	505 (49.41)	627 (44.59)	
Medication at admission, *n* (%)							
Antihypertensive drugs	10,773	4,886 (45.35)	1,780 (40.47)	1,786 (45.25)	540 (52.84)	780 (55.48)	<0.0001
Glucose-lowing drugs	10,773	2,023 (18.78)	729 (16.58)	712 (18.04)	258 (25.24)	324 (23.04)	<0.0001
Antiplatelet drugs	10,773	1,938 (17.99)	601 (13.67)	612 (15.51)	269 (26.32)	456 (32.43)	<0.0001
Lipid-lowing drugs	10,773	1,262 (11.71)	391 (8.89)	387 (9.80)	188 (18.40)	296 (21.05)	<0.0001
Blood pressure at admission							
SBP (mmHg)	10,773	148.50 (135.00–164.00)	147.50 (135.00–163.50)	149.00 (135.00–165.00)	150.00 (134.5–165.00)	147.50 (135.00–162.50)	0.1810
DBP (mmHg)	10,773	86.50 (79.00–95.00)	87.50 (80.00–96.50)	86.00 (79.00–95.00)	86.50 (79.00–95.00)	84.00 (77.50–92.00)	<0.0001
BMI (IQR)	10,773	24.49 (22.58–26.57)	24.49 (22.66–26.57)	24.24 (22.49–26.51)	24.76 (23.03–26.73)	24.49 (22.58–26.67)	0.0015
Biochemical markers, median (IQR)							
Glucose (mmol/L)	8,594	5.53 (4.90–6.90)	5.41 (4.86–6.57)	5.60 (4.90–6.98)	5.70 (4.99–7.39)	5.77 (4.97–7.37)	<0.0001
TC (mmol/L)	10,713	4.00 (3.34–4.77)	4.07 (3.40–4.81)	3.94 (3.28–4.72)	4.09 (3.42–4.86)	3.89 (3.23–4.65)	<0.0001
TG (mmol/L)	10,708	1.37 (1.03–1.89)	1.42 (1.06–1.96)	1.32 (1.02–1.82)	1.41 (1.04–1.94)	1.30 (0.97–1.79)	<0.0001
LDL (mmol/L)	10,705	2.35 (1.75–3.01)	2.37 (1.77–3.04)	2.32 (1.74–2.97)	2.39 (1.78–3.12)	2.32 (1.70–2.96)	0.0263
HDL (mmol/L)	10,703	1.08 (0.90–1.28)	1.10 (0.94–1.31)	1.06 (0.88–1.26)	1.09 (0.92–1.30)	1.04 (0.87–1.24)	<0.0001
IL-6 (pg/ml)	10,773	2.64 (1.58–4.98)	1.57 (1.17–2.05)	4.88 (3.43–8.48)	1.69 (1.25–2.08)	5.37 (3.66–9.75)	<0.0001

*PolyVD, polyvascular disease; IQR, interquartile range; NHISS, National Institutes of Health Stroke Scale; TOAST, Trial of Org 10172 in Acute Stroke Treatment; LAA, large artery atherosclerosis; CE, cardioembolism; SVO, small-vessel occlusion; SBP, systolic blood pressure; DBP, diastolic blood pressure; BMI, body mass index; TC, total cholesterol; TG, triglyceride; LDL, low-density lipoprotein; HDL, high-density lipoprotein; IL-6, interleukin-6*.

### Combination of PolyVD With IL-6 and Outcomes

Overall, the cumulative incidence of recurrent stroke and MACEs was 9.87 and 10.44%, respectively, during the 1-year follow-up. Among all the patients with recurrent stroke, 178 (12.66%), 109 (10.67%), 434 (11.0%), and 338 (7.69%) had PolyVD with IL-6 ≥ 2.64 pg/mL, PolyVD with IL-6 < 2.64 pg/ml, non-PolyVD with IL-6 ≥ 2.64 pg/ml, and non-PolyVD with IL-6 < 2.64 pg/ml, respectively (Table 2).

**Table 2:** Clinical outcomes of patients included at 1 year follow-up.

**Clinical outcomes**	**Overall**	**non-PolyVD, IL-6 <2.64 pg/ml**	**non-PolyVD, IL-6 ≥ 2.64 pg/ml**	**PolyVD, IL-6 <2.64 pg/ml**	**PolyVD, IL-6 ≥ 2.64 pg/ml**	***P*-value**
Recurrent stroke	1059 (9.87)	338 (7.69)	434 (11.0)	109 (10.67)	178 (12.66)	<0.0001
MACEs	1120 (10.44)	346 (7.87)	455 (11.53)	119 (11.64)	200 (14.22)	<0.0001
All-cause mortality	368 (3.43)	54 (1.23)	174 (4.41)	25 (2.45)	115 (8.18)	<0.0001
Poor functional outcomes (mRS 3–6)	1423 (13.26)	307 (6.98)	695 (17.61)	86 (8.41)	335 (23.83)	<0.0001

*PolyVD, polyvascular disease; IL-6, interleukin-6; MACEs, major adverse cardiovascular events; mRS, modified Rankin scale*.

**Figure 2:**
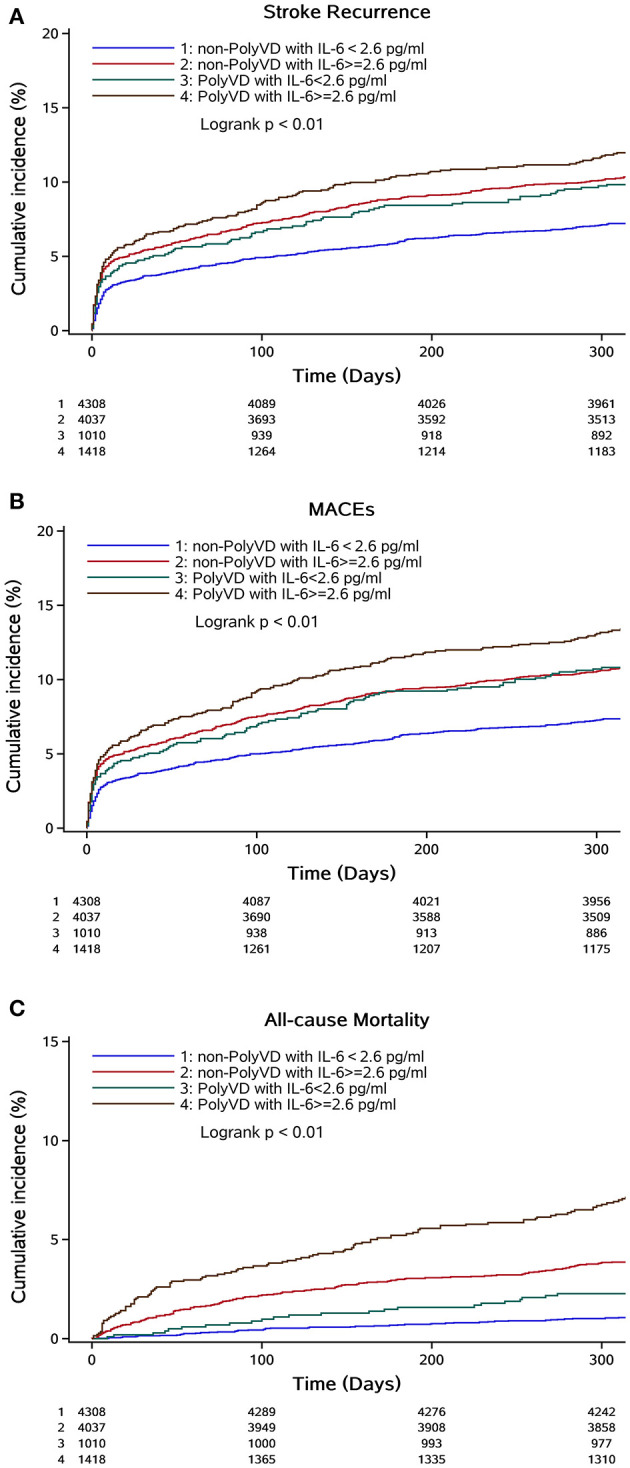
Cumulative incidence of recurrent stroke, MACEs, and all-cause mortality at 1-year follow-up based on atherosclerosis vascular beds involvement numbers and IL-6 level. **(A–C)** Kaplan–Meier curves of incidence of recurrent stoke, MACEs, and all-cause mortality within 1 year, respectively.

The association of the combined effect of vascular beds involvement numbers and IL-6 levels with clinical outcomes at 1-year follow-up by COX proportional hazard model is shown in Table 3. After adjusting for age, gender, education, current smoking, heavy drinking, medical history of hypertension, DM, dyslipidemia, IS, AF, infection, NIHSS at admission, the subtype of stroke, medication and DBP (mmHg), BMI (mean±SD), glucose (mmol/L), TC (mmol/L), TG (mmol/L), LDL (mmol/L), and HDL (mmol/L), patients with PolyVD and IL-6 ≥ 2.64 pg/mL had an increased risk of recurrent stroke at 1-year follow-up compared to those with non-PolyVD and IL-6 < 2.64 pg/ml, the adjusted HR (95% CI) was 1.290 (1.058–1.572); *P* = 0.01. Moreover, compared to patients with non-PolyVD and IL-6 < 2.64 pg/ml, those with non-PolyVD with IL-6 ≥ 2.64 pg/ml (HR: 1.245, 95% CI 1.072–1.446; *P* = 0.004) and PolyVD with IL-6 < 2.64 pg/ml (HR: 1.251, 95% CI: 1.002–1.563; *P* = 0.04) were associated with an increased risk of recurrent stroke within 1 year after adjusting for the confounding factors. Kaplan–Meier curves of the PolyVD and IL-6 groups appeared distinctly early in the first year, indicating that patients with PolyVD and IL-6 ≥ 2.64 pg/ml had the highest incidence of recurrent stroke (Figure 2). Similar results were observed for MACEs.

**Table 3:** HR (95% CIs) of clinical outcomes according to atherosclerosis vascular beds involvement numbers and IL-6 level in patients with AIS or TIA at 1 year follow-up.

**Clinical outcomes**	**Group**	**Unadjusted model**		**Adjusted model 1**		**Adjusted model 2**	
		**HR (95% CI)**	***P*-value**	**HR (95% CI)**	***P*-value**	**HR (95% CI)**	***P*-value**
Recurrent stroke	non-PolyVD, IL-6 <2.64 pg/ml	1 (Reference)	-	1 (Reference)	-	1 (Reference)	-
	non-PolyVD, IL-6≥2.64 pg/ml	1.502 1.302–1.732	<0.0001	1.263 1.088–1.466	0.0022	1.245 1.072–1.446	0.0041
	PolyVD, IL-6 <2.64 pg/ml	1.425 1.145–1.772	0.0015	1.265 1.013–1.579	0.0382	1.251 1.002–1.563	0.0479
	PolyVD, IL-6≥2.64 pg/ml	1.759 1.466–2.110	<0.0001	1.303 1.070–1.586	0.0084	1.290 1.058–1.572	0.0118
MACEs	non-PolyVD, IL-6 <2.64 pg/ml	1 (Reference)	-	1 (Reference)	-	1 (Reference)	-
	non-PolyVD, IL-6≥2.64 pg/ml	1.536 1.335–1.768	<0.0001	1.281 1.107–1.483	0.0009	1.264 1.091–1.464	0.0018
	PolyVD, IL-6 <2.64 pg/ml	1.523 1.234–1.880	<0.0001	1.341 1.083–1.662	0.0072	1.327 1.071–1.645	0.0096
	PolyVD, IL-6≥2.64 pg/ml	1.933 1.623–2.302	<0.0001	1.406 1.164–1.699	0.0004	1.389 1.148–1.680	0.0007
All-cause mortality	non-PolyVD, IL-6 <2.64 pg/ml	1(Reference)	-	1 (Reference)	-	1 (Reference)	-
	non-PolyVD, IL-6≥2.64 pg/ml	3.770 2.761–5.147	<0.0001	2.075 1.501–2.867	<0.0001	2.089 1.511–2.890	<0.0001
	PolyVD, IL-6 <2.64 pg/ml	2.106 1.305–3.398	0.0023	1.614 0.995–2.620	0.0525	1.600 0.985–2.597	0.0574
	PolyVD, IL-6≥2.64 pg/ml	7.120 5.120–9.902	<0.0001	3.069 2.153–4.376	<0.0001	3.116 2.180–4.452	<0.0001
Poor functional outcomes (mRS 3-6)	non-PolyVD, IL-6 <2.64 pg/mL	1 (Reference)	-	1 (Reference)	-	1 (Reference)	-
	non-PolyVD, IL-6≥2.64 pg/ml	2.857 2.476–3.297	<0.0001	1.534 1.311–1.795	<0.0001	1.533 1.308–1.796	<0.0001
	PolyVD, IL-6 <2.64 pg/ml	1.209 0.938–1.558	0.1422	0.895 0.686–1.168	0.4137	0.885 0.677–1.156	0.3687
	PolyVD, IL-6≥2.64 pg/ml	4.226 3.567–5.008	<0.0001	1.726 1.419–2.101	<0.0001	1.743 1.430–2.124	<0.0001

*Model 1: Adjusted for age, gender, education, current smoking, heavy drinking, hypertension, DM, dyslipidemia, AF, previous IS, NIHSS score at admission, subtype of stroke, medication at admission, and infection*.

*Model 2: Adjusted variables in model 2 and plus DBP (mmHg), BMI (mean ± SD), glucose (mmol/L), TC (mmol/L), TG (mmol/L), LDL (mmol/L), and HDL (mmol/L)*.

*PolyVD, polyvascular disease; IL-6, interleukin-6; AIS, acute ischemic stroke; TIA, transient ischemic attack; MACEs, major adverse cardiovascular events. HR, hazard ratio; NHISS, National Institutes of Health Stroke Scale; DBP, diastolic blood pressure; BMI, body mass index; TC, total cholesterol; TG, triglyceride; LDL, low-density lipoprotein; HDL, high-density lipoprotein*.

All-cause mortality and poor functional outcomes (mRS 3–6) were recorded in 368 (3.43%) and 1,423 (13.26%) patients within 1-year follow-up (Table 2). In a fully adjusted model, compared to non-PolyVD and IL-6 < 2.64 pg/ml patients, PolyVD with IL-6 ≥ 2.64 pg/ml (HR: 1.743, 95% CI: 1.430–2.124; *P* < 0.001) and non-PolyVD with IL-6 ≥ 2.64 pg/ml (HR: 1.533, 95% CI: 1.308–1.796; *P* < 0.001) patients had an increased risk of poor functional outcomes at 1-year follow-up. Similar results were observed for death (Table 3).

## Discussion

This study demonstrated that PolyVD together with the elevated IL-6 levels increased the risk of stroke recurrence, MACEs, all-cause mortality, and poor functional outcomes in patients with AIS or TIA at 1-year follow-up. Thus, combined PolyVD and elevated IL-6 levels could increase the stroke risk stratification efficiency compared with when used alone.

CAD, CVD, and PAD are common atherosclerotic diseases associated with high morbidity and mortality worldwide (21). PolyVD is widespread in patients with different diseases and varied prevalence. In the Can Rapid Risk Stratification of Unstable Angina Patients Suppress Adverse Outcomes with Early Implementation of the ACC/AHA Guidelines (CRUSADE) study, PolyVD occurred in about 13% of the patients with CAD (22). In addition, the Examining Use of Ticagrelor in Peripheral Artery Disease (EUCLID) trial reported that PolyVD occurred in 43.8% of the patients with PAD (23). Herein, we focused on PolyVD in patients with CVD. The MITICO study reported that in ischemic stroke patients, who did not receive any anti-coagulant treatment, PolyVD occurred in about 14.0% of the patients (24). Furthermore, in Stroke in Italy and Related Impact on Outcome (SIRIO) trial, PolyVD occurred in 32.1% of the patients with IS (25), while in the Reduction of Atherothrombosis for Continued Health (REACH) Registry, about 40.1% of CVD patients had PolyVD (26). In the current study, 22.5% of the AIS or TIA patients had PolyVD which was consistent with other studies incorporating the similar population from different countries (24–26).

Compared to patients with single vascular bed injury, patients with PolyVD were associated with a high risk of MACEs. The MITICO study demonstrated that IS patients with PolyVD showed high rates of vascular recurrence (24). The SIRIO trial also reported that in patients with AIS, PolyVD status significantly affected the mortality and MACEs after discharge (OR: 1.44; 95% CI: 1.10–1.88) (25). In this study, it is also observed that the risk of recurrent stroke and MACEs increased significantly in patients with PolyVD, compared to those with non-PolyVD and IL-6 <2.64 pg/ml. Intriguingly, the risk of MACEs was also elevated with the increase in the injury of vascular bed numbers (11). The REACH registry reported that the risk of MACEs had gradually increased from 4.1% in single vascular bed to 9.2% in patients with three vascular beds damaged at 1-year follow-up (27).

Compared to single vascular beds damage, PolyVD patients had a higher risk of recurrent vascular events and worse outcomes, which might be related to a greater extension of atherosclerosis and a higher risk of unstable plaques (28, 29). Accumulating evidence has confirmed that inflammatory cells and factors play critical roles in promoting atherosclerosis, thereby destabilizing the atherosclerotic plaque, platelet aggregation, and intravascular thrombosis, which increased the risk of stroke (30, 31). As an upstream and potent inflammatory factor, IL-6 was closely related to adverse stroke outcomes. Several studies showed that an elevated level of IL-6 was associated with brain infarction volume, stroke severity, and prognosis of IS (32–35). Also, IL-6 was strongly associated with thin-cap fibroatheroma during percutaneous coronary intervention, indicating a potential mechanistic correlation with rupture-prone plaques (36). In the current study, it is found that compared to patients with non-PolyVD and IL-6 <2.64 pg/ml, patients with elevated IL-6 levels had the higher risk of recurrent stroke and MACEs, especially patients also with PolyVD.

PolyVD and elevated IL-6 levels are related to poor outcomes of IS (16–18, 24, 25). Previous studies reported that the level of IL-6 was higher in PolyVD patients (24, 37); however, only a few studies focused on their combined effect on the outcomes. In this study, it is observed that patients with PolyVD or patients with elevated IL-6 levels were all associated with increased risk of recurrent stroke compared with patients with non-PolyVD and IL-6 <2.64 pg/ml, however, AIS/TIA patients with both PolyVD and elevated IL-6 levels had the highest risk of recurrent stroke among groups, indicating that PolyVD and plaque instability had a combined effect on adverse outcomes. Thus, a correlation between vascular bed injury numbers and plaque stability could be established, which had an effect on stroke outcomes. According to the data from MITICO study, stroke patients with polyvascular atherothrombotic disease showed a high rate of vascular recurrence and robust association with inflammatory markers (24). Another study comprising of 3,007 patients with a recently symptomatic carotid stenosis demonstrated that patients with unstable plaques in one carotid artery were rather prone to irregular plaques in the other carotid artery. These patients were likely to have CAD and suffer from subsequent vascular events (38). However, the specific mechanism underlying the combined effect of PolyVD and IL-6 on IS outcomes need further investigation.

Additionally, we investigated the association of combined PolyVD and IL-6 with all-cause mortality and functional outcomes that have been reported scarcely. A meta-analysis of 24 studies included 4,112 stroke patients with IL-6 levels in the 4th quartile that were independently associated with poor functional outcomes in the short-term follow-up (39). Some recent studies also confirmed that a high level of IL-6 after AIS was an independent predictor of poor functional outcomes (18, 35, 40). The current findings also supported that patients with PolyVD and elevated levels of IL-6 had the highest risk of mortality and poor functional outcomes compared to other groups. Interestingly, patients with non-PolyVD and elevated levels of IL-6 also had a moderate risk of death and poor functional outcomes, while patients with PolyVD and non-elevated level of IL-6 were not independently associated with death and poor functional outcomes in the fully adjusted model. This finding suggested that IL-6 might partially contribute to death and poor functional outcomes by stimulating acute-phase reactants, activating endothelial cells, and disrupting the plaque stability (15).

## Limitations

Although this was a multicenter cohort study, including large sample and centralized detection of blood samples, several limitations cannot be ignored. First, we did not routinely screen PAD in all patients, and the diagnosis of PolyVD was mainly based on the patients' medical history, and patients with asymptomatic or mild symptoms might have been ignored without a timely diagnosis. Second, some patients in CNSRIII without the estimation of IL-6 were excluded from the study. Third, additional inflammatory markers and other aspects of plaque stability were not assessed. Fourth, we only measured the baseline level of IL-6 and did not analyze the clinical outcomes of stroke with respect to the dynamic changes in IL-6 at different time points.

## Conclusions

PolyVD and elevated IL-6 levels are both associated with poor outcomes in patients with AIS or TIA. Moreover, the combination of them increases the efficiency of stroke risk stratification compared with when used alone. More attention and intensive treatment should be given to those patients with both PolyVD and elevated IL-6 levels.

## Data Availability Statement

The original contributions presented in the study are included in the article/Supplementary Material, further inquiries can be directed to the corresponding author/s.

## Ethics Statement

The studies involving human participants were reviewed and approved by Ethics Committees of Beijing Tiantan Hospital. The patients/participants provided their written informed consent to participate in this study.

## Author Contributions

LG and XM had full access to all of the data in the study and take responsibility for the integrity of the data and the accuracy of the data analysis. YT, JJ, LG, XM, and YW helped in study concept and design and drafting of the manuscript. YT, JJ, AW, YZ, YJ, JL, XZ, HL, HW, and YW contributed to acquisition, analysis, or interpretation of the data. Statistical analysis was performed by AW and YZ. All authors contributed to manuscript revision, read, and approved the submitted version.

## Conflict of Interest

The authors declare that the research was conducted in the absence of any commercial or financial relationships that could be construed as a potential conflict of interest.
