# Supplementary material for: Association of Polyvascular Disease and Elevated Interleukin-6 With Outcomes in Acute Ischemic Stroke or Transient Ischemic Attack
Source: Front Neurol. 2021 Apr 13;12:661779. doi: 10.3389/fneur.2021.661779 (PMC8076541; doi:10.3389/fneur.2021.661779)
Supplement: Supplementary file 1 [file Table_1.pdf]

Supplementary Table 1. Comparison between patients included and patients excluded.

Characteristics	Patients included (n=10773)	Patients excluded (n=4393)	*P*-value
Age (mean±SD), y	62.28±11.31	62.11±11.29	0.3934
Male sex, n (%)	7376 (68.47)	2988 (68.02)	0.5888
Education			<0.0001
Elementary or below	2919 (27.10)	1373 (31.25)	
Middle school	3158 (29.31)	1247 (28.39)	
High school or above	3188 (29.59)	1094 (24.90)	
Unknown	1508 (14.00)	679 (15.46)	
Current smoking	3404 (31.60)	3045 (69.31)	0.2719
Heavy drinking	1531 (14.21)	595 (13.54)	0.2830
Hypertension	6743 (62.59)	2751 (62.62)	0.9717
Diabetes mellitus	2586 (24.00)	924 (21.03)	<0.0001
Dyslipidemia	905 (8.40)	286 (6.51)	<0.0001
Atrial fibrillation	762 (7.07)	257 (5.85)	0.0064
Previous ischemic stroke	2432 (22.57)	923 (21.01)	0.0353
Infection, n (%)	308 (2.86)	142 (3.23)	0.1081
NIHSS score at admission, median(IQR)	3 (1–6)	3 (1–6)	0.0083
Subtype of stroke (TOAST)			0.0352
LAA	2692 (24.99)	1164 (26.50)	
CE	684 (6.35)	233 (5.30)	
SVO	2258 (20.96)	907 (20.65)	
Other causes	5139 (47.70)	2089 (47.55)	
Medication at admission, n (%)			
Antihypertensive drugs	4886 (45.35)	1909 (43.46)	0.0329
Glucose-lowing drugs	2023 (18.78)	750 (17.07)	0.0137
Antiplatelet drugs	1938 (17.99)	667 (15.18)	<0.0001
Lipid-lowing drugs	1262 (11.71)	409 (9.31)	<0.0001
Blood pressure at admission			
SBP (mmHg)	148.50 (135.00–164.00)	148.00 (135.00–162.5)	0.2762
DBP (mmHg)	86.50 (79.00–95.00)	86.00 (79.00–95.50)	0.7418
BMI (IQR)	24.49 (22.58–26.57)	24.49 (22.68–26.44)	0.8521
Biochemical markers, median (IQR)			
Glucose(mmol/L)	5.53 (4.90–6.90)	5.50 (4.88–6.86)	0.1774
TC (mmol/L)	4.00 (3.34–4.77)	4.49 (3.81–5.25)	<0.0001
TG (mmol/L)	1.37 (1.03–1.89)	1.39 (1.01–1.96)	0.2894

LDL (mmol/L)	2.35 (1.75–3.01)	2.70 (2.12–3.32)	<0.0001
HDL (mmol/L)	1.08 (0.90–1.28)	1.10 (0.93–1.32)	<0.0001

Abbreviations: IQR, interquartile range; NHISS, National Institutes of Health Stroke Scale; TOAST, Trial of Org 10172 in Acute Stroke Treatment; LAA, large artery atherosclerosis; CE, cardioembolism; SVO, small-vessel occlusion; SBP, systolic blood pressure; DBP, diastolic blood pressure; BMI, body mass index; TC, total cholesterol; TG, triglyceride; LDL, low-density lipoprotein; HDL, high-density lipoprotein.
